# 95 Parental education, physical activity, sedentary behavior and Family Health Climate of children in primary-school age: A mediation analysis

**DOI:** 10.1093/eurpub/ckae114.093

**Published:** 2024-09-26

**Authors:** Alexandra Ziegeldorf, Nina Hottenrott, Johanna Moritz, Petra Wagner, Hagen Wulff

**Affiliations:** Leipzig University, Leipzig, Germany; Leipzig University, Leipzig, Germany; Leipzig University, Leipzig, Germany; Leipzig University, Leipzig, Germany; Leipzig University, Leipzig, Germany

## Abstract

**Purpose:**

Parents’ level of education (ED) is an important factor influencing children’s physical ac-tivity (PA) and sedentary behaviour (SED). In this context, family factors, such as the physical activity related family health climate (FHCPA), are relevant (Niermann et al., 2015). However, the effect of FHCPA on the interaction between parental ED and chil-dren’s PA and SED has not been investigated yet. In this analysis, the mediating effect of the FHCPA on the relation between ED and objectively measured PA-parameters was examined.

**Methods:**

For this analysis, data from 94 children (51.1% female, Mean age = 9,0 (±1)) and their parents from the collaborative research project “Familie+” (Baseline) have been used. Questionnaires were used to assess parental ED and FHCPA. Children’s moderate-to-vigorous physical activity (MVPA) and SED were measured using accelerometers (GT3X+ und wGT3X+). Bivariate correlations were conducted to investigate associations between parental ED and MVPA/SED/FHCPA. Mediation analyses were used to investigate the role of FHCPA in the link between maternal and paternal ED and children’s MVPA/SED.

**Results:**

Between maternal ED and FHCPA there was a small correlation for the total sample (ρ=.318, p<.001) and a medium correlation for girls only (ρ=.570, p<.001). Mediation analyses showed no significant mediation effect. However, there was a significant direct association considering FHCPA in the relation between higher maternal ED and SED in girls compared to lower ED.

**Conclusion:**

Future research should examine more complex models to develop and sharpen further theoretical frameworks in order to be able to derive better recommendations through health prevention programs, especially for mothers and girls.

**Support/Funding Source:**

The data originate from a collaborative research project (‘Familie+ - Gesundes Zusam-menleben in Familie und Schule’/‘Family+ - Living healthily together in family and school’) which was funded by the German Federal Ministry of Health.

**References:**

Niermann et al. (2015). Family Health Climate and Adolescents `Physical Activity and Healthy Eating: A Cross-Sectional Study with Mother-Father-Adolescent Triads. PLoS ONE 10 (11). 1-18.

